# Impact of gastro-oesophageal reflux on microRNA expression, location and function

**DOI:** 10.1186/1471-230X-13-4

**Published:** 2013-01-08

**Authors:** Cameron M Smith, Michael Z Michael, David I Watson, Grace Tan, David St J Astill, Richard Hummel, Damian J Hussey

**Affiliations:** 1 Department of Surgery, Flinders University, Room 3D213, Flinders Medical Centre, Bedford Park, South Australia, 5042, Australia; 2 Department of Gastroenterology & Hepatology, Flinders University, Bedford Park, South Australia, Australia; 3 Flinders Centre for Cancer Prevention and Control, Flinders University, Bedford Park, South Australia, Australia; 4 Department of Anatomical Pathology, Flinders Medical Centre, Bedford Park, South Australia, 5042, Australia

**Keywords:** microRNA, Gastro-oesophageal reflux disease, Ulcerative oesophagitis, Apoptosis, Proliferation, Barrett’s oesophagus

## Abstract

**Background:**

Ulceration of the oesophageal squamous mucosa (ulcerative oesophagitis) is a pathological manifestation of gastro-oesophageal reflux disease, and is a major risk factor for the development of Barrett’s oesophagus. Barrett’s oesophagus is characterised by replacement of reflux-damaged oesophageal squamous epithelium with a columnar intestinal-like epithelium. We previously reported discovery of microRNAs that are differentially expressed between oesophageal squamous mucosa and Barrett’s oesophagus mucosa. Now, to better understand early steps in the initiation of Barrett’s oesophagus, we assessed the expression, location and function of these microRNAs in oesophageal squamous mucosa from individuals with ulcerative oesophagitis.

**Methods:**

Quantitative real-time PCR was used to compare miR-21, 143, 145, 194, 203, 205 and 215 expression levels in oesophageal mucosa from individuals without pathological gastro-oesophageal reflux to individuals with ulcerative oesophagitis. Correlations between microRNA expression and messenger RNA differentiation markers *BMP-4, CK8* and *CK14* were analyzed. The cellular localisation of microRNAs within the oesophageal mucosa was determined using in-situ hybridisation. microRNA involvement in proliferation and apoptosis was assessed following transfection of a human squamous oesophageal mucosal cell line (Het-1A).

**Results:**

miR-143, miR-145 and miR-205 levels were significantly higher in gastro-oesophageal reflux compared with controls. Elevated miR-143 expression correlated with *BMP-4* and *CK8* expression, and elevated miR-205 expression correlated negatively with *CK14* expression. Endogenous miR-143, miR-145 and miR-205 expression was localised to the basal layer of the oesophageal epithelium. Transfection of miR-143, 145 and 205 mimics into Het-1A cells resulted in increased apoptosis and decreased proliferation.

**Conclusions:**

Elevated miR-143, miR-145 and miR-205 expression was observed in oesophageal squamous mucosa of individuals with ulcerative oesophagitis. These miRNAs localised to the basal layer of the oesophageal epithelium. They reduced proliferation and increased apoptosis, and may play roles in regulating epithelial restoration in response to injury caused by gastro-oesophageal reflux.

## Background

Gastro-oesophageal reflux disease affects up to 50% of Western populations [1]. Gastric physiology offers protection from exposure to acid and bile, although the oesophageal lumen is largely unprotected, lacking an adherent mucous barrier [2]. Patients with chronic reflux can present with endoscopically visible mucosal damage, and this manifests as mucosal ulceration (ulcerative oesophagitis) [1]. At the cellular level, ulcerative oesophagitis is associated with increased cell proliferation [3] and apoptosis [4]. Increased inflammatory cell infiltrate [5] and hyper-proliferation of basal cells are typical histopathological changes associated with gastro-oesophageal reflux [3,6]. We have shown previously that increases in cytokeratin (CK) 14 messenger RNA (mRNA) levels correlate with increased severity of reflux [1]. This appears to be a molecular marker of basal cell hyperplasia.

Ulcerative oesophagitis is a major independent risk factor for the development of Barrett’s oesophagus [7], which is characterised by a metaplastic transition from normal oesophageal squamous epithelium to a columnar epithelium with intestinal features [2]. Barrett’s oesophagus is associated with a 40 fold increase in the risk of developing oesophageal adenocarcinoma, a cancer with poor prognosis [8]. Although Barrett’s oesophagus is not a cancerous lesion, the increased risk of cancer development it carries means that investigating markers associated with the development of Barrett’s oesophagus is worthwhile. Studying gene expression in squamous epithelium that has been exposed to reflux might identify changes that precede progression to Barrett’s oesophagus. Previous reports have shown increased expression of the *BMP-4* gene, in squamous oesophageal epithelium from individuals with ulcerative oesophagitis [9]. Further, reflux induced expression of BMP-4 has been shown to induce a shift from a squamous to a columnar gene expression profile in oesophageal squamous cells [9,10]. This suggests that reflux induced gene expression changes in ulcerative oesophagitis may promote conversion from squamous to columnar epithelium.

MicroRNAs (miRNAs) are small RNA molecules that regulate gene expression [11]. Preliminary evidence implicates miRNAs in the differentiation of intestinal [12] and squamous epithelial cell phenotypes [13], and cell proliferation and apoptosis [14]. We have identified miRNAs that are differentially expressed in Barrett’s oesophagus [15]. miR-203 and miR-205 are expressed at higher levels in squamous mucosa, and miR-143, miR-145, miR-194 and miR-215 are expressed at higher levels in Barrett’s oesophagus. Currently, it is not known whether miRNA expression is altered in gastro-oesophageal reflux exposed squamous epithelium at risk of progressing to Barrett’s oesophagus. If miRNA expression is altered in the squamous epithelium of individuals with ulcerative oesophagitis, then this might provide insight into the development of Barrett’s oesophagus.

In this study we hypothesised that miR-203, 205, 143, 145, 194 and 215 expression might be altered in esophageal squamous mucosa in response to chronic gastro-oesophageal reflux. The aims of the study were to 1) Determine the expression of miRNAs known to be differentially expressed between normal squamous and Barrett’s oesophagus mucosa (miR-203, 205, 143, 145, 194 and 215) in oesophageal squamous mucosa from individuals with ulcerative oesophagitis; 2) Assess the location of miRNA accumulation within the oesophageal epithelium; 3) Assess the role of these miRNAs in regulating proliferation and apoptosis, processes that contribute to tissue restoration in reflux-damaged mucosa.

## Methods

### Tissue collection and processing

Oesophageal mucosal fresh tissue biopsies were collected from individuals undergoing upper gastrointestinal endoscopy. Full details of the tissue collection protocol and RNA extraction procedure have been described previously [1,15]. In the current study we used mucosal biopsies collected from the oesophageal squamous mucosa 5 cm above the gastro-oesophageal junction from normal subjects and from subjects with gastro-oesophageal reflux with ulcerative oesophagitis. Normal subjects without clinical or endoscopic evidence of gastro-oesophageal reflux were used as the controls (n=13; median age 54 years). Selection criteria for controls were a visibly normal oesophageal mucosa at endoscopy, no endoscopic indicators of mechanical incompetence of the gastro-oesophageal junction, no current or previous symptoms of gastro-oesophageal reflux disease and no use of antisecretory medication. Subjects with gastro-oesophageal reflux (n=10; median age 52 years) were included if they had typical symptoms of gastro-oesophageal reflux disease (heartburn and regurgitation), mucosal ulceration (Savary Miller grade I to IV) at endoscopy, and no endoscopic or histological evidence of Barrett’s oesophagus. The Flinders Clinical Research Ethics Committee approved tissue collection.

Mucosal biopsies were collected and stored in RNAlater® (Ambion, Austin, Texas, USA) at −20°C. When required, biopsies were thawed and a small piece of each biopsy was fixed using formalin, embedded in paraffin for histopathological confirmation of the tissue origin. RNA was extracted from once thawed tissue using *TRI*zol® as described previously [1]. RNA yield was determined by spectrophotometry, and the quality assessed by agarose gel electrophoresis. Oesophageal mucosal tissue biopsies, used for in-situ hybridisation, were collected adjacent to those used to assess miRNA and mRNA expression by the same collection protocol described above. These biopsies were fixed immediately with 10% formalin and subsequently embedded in paraffin.

### miRNA and mRNA quantitation by real-time RT-PCR

Quantitative real-time PCR was used to measure miRNA and mRNA expression levels in oesophageal mucosal biopsies from individuals without pathological reflux and individuals with ulcerative oesophagitis. The expression of miRNAs in the different types of oesophageal squamous mucosa was determined using previously described methods [15]. miR-21 (P/N# 4373090), miR-143 (P/N# 4373134), miR-145 (P/N# 4373133), miR-194 (P/N# 437310), miR-203 (P/N# 4373095), miR-205 (P/N# 4373093), miR-215 (P/N# 4373084) levels were determined by qRT-PCR using TaqMan® miRNA assays (Applied Biosystems, Foster City, California, USA). Due to limited RNA, miR-145 expression was only assessed in ten of the 13 controls*.* The levels of differentially expressed miRNAs were correlated with the mRNA expression levels of squamous (*CK14*) and columnar (*CK8* and *BMP-4*) differentiation markers in squamous mucosa from patients with gastro-oesophageal reflux. mRNA levels were assessed using a Quantiscript® RT kit for reverse transcription and the Quantitect® SYBRGreen mastermix for PCR (Qiagen, Valencia, California). Primer details are available upon request. miRNA expression was normalised to U44 small nucleolar RNA levels (P/N# 4373384) and mRNA expression was normalised to ß-actin levels.

### In-Situ Hybridisation (ISH)

To localise miRNA activity within the oesophageal mucosa in-situ hybridisation was performed, probing against miRNAs elevated in ulcerative oesophagitis. Standard hematoxylin and eosin (H&E) staining was performed to confirm the tissue type. In-situ hybridisation was performed on serial 4 μm sections of 4% formalin fixed, paraffin embedded oesophageal tissue biopsies from controls and individuals with ulcerative oesophagitis. The in-situ hybridisation protocol was based on a protocol published by Pena and colleagues [16] with the following changes. All buffers were prepared with diethyl pyrocarbonate (DEPC) treated water (Sigma, Castle Hill, NSW) unless otherwise stated. Sections were deparaffinised in Histo-Clear (National Diagnostics, Atlanta, Georgia) and rehydrated through an ethanol dilution series (100-25%). Slides were then washed for 5 min in water treated with DEPC followed by 13 min in 2 μg/ml proteinase K treatment (Roche Diagnostics, Mannheim, Germany). Tissue sections were acetylated for 10 min and then washed twice, for five minutes in PBS. Sections were framed with a wax pen (Vector Laboratories, Burlingame, California), covered with 40 μl of pre-hybridisation solution [16] and incubated at room temperature for four hours. Pre-hybridisation buffer was tipped from the slides and replaced with 40 μl of either the digoxigenin (DIG)-labeled, Locked Nucleic Acid (LNA)-miRNA probe or LNA-scramble probe (Exiqon, Vedbaek, Denmark) diluted to 250 nM with denaturing hybridisation buffer (0.25% CHAPS (Amresco, Solon #0465-5g), 0.1% Tween-20 (Sigma-Aldrich, St Louis, MO, USA #P1379). Slides were then incubated at 55°C overnight. The LNA-probes used include miR-143 (#38515-15), miR-145 (#38517-15), miR-205 (#18099-15) and miR-SCR (#99004-15). The sequence of the miR-SCR duplex, used as a negative control, shared no homology with known miRNAs.

Slides were washed twice with 0.2X SSC buffer heated to 60°C and then incubated with 0.2X SSC buffer 60 min at 60°C. Levamisole (24% w/v) (Sigma Aldrich, St Louis, Mo, USA, #L9756-5G) was used to reduce background alkaline phosphate activity. Slides were submerged in levamisole buffer containing 1M TRIS pH 7.5 and 5M NaCl for 5 min at room temperature. The sections were then incubated with a blocking buffer (1% blocking buffer and 1X maleic acid buffer, DIG Wash and Block Buffer Set, (Roche Diagnostics, Mannheim, Germany) for 60 min. 40 μl of anti-DIG antibodies (Roche Diagnostics, Mannheim, Germany) diluted 1:2000 using blocking buffer was added to each section and then slides were incubated overnight at 4°C. The slides were then washed in 1X wash buffer (DIG Wash and Block Buffer Set, Roche Diagnostics, Mannheim, Germany) and incubated with the detection buffer (0.08 M Tris-Cl containing 0.17 M NaCl pH 9.5) for 10 min. The slides were then submerged in a 1:500 dilution of nitro blue tetrazolium chloride (NBT) /BCIP (5-bromo-4-chloro-3-indolyl phosphate, toluidine salt) (Roche Diagnostics, Mannheim, Germany) until staining was observed. Buffered glycerol was added to each section before cover-slipping.

### Het-1A Cell Culture and miRNA over-expression

To assess the impact of selected miRNAs on proliferation and apoptosis in oesophageal epithelial cells miR-143, miR-145 or miR-205 were overexpressed in Het-1A cells, a non-neoplastic oesophageal keratinocyte derived cell line [1,17]. Het-1A cells were cultured using LHC-9 medium (Invitrogen, Mulgrave, Victoria). Cells were transfected with either miR-143, miR-145 or miR-205 mimics or a negative control duplex. Experiments measuring cell proliferation were conducted using 96 well plates. Three thousand Het-1A cells were plated per well and 10 wells were used for each experimental and control group. Experiments measuring apoptosis were conducted using 24 well plates, 50,000 Het-1A cells were plated per well and 8 wells were used for each experimental and control group.

miRNA levels were increased by transfecting cells with miR-143, miR-145 or miR-205 mimics using the Lipofectamine™ 2000 system (Invitrogen, Mulgrave, Victoria) as per the manufacturer’s protocol. Cells were transfected using miRNA mimic or negative control duplexes at 33 nM concentration (miR-143 #2988, miR-145 #8480, miR-205 #3391, negative control duplex #1733 all from GenePharma, Shanghai, China).

Cell proliferation and apoptosis were assayed, and miRNA over-expression was confirmed 24 and 48 h post transfection. Cell numbers were measured using the MTS assay system (Invitrogen, Mulgrave, Victoria) as per the manufacturer’s protocol. Cell numbers at each time point were assessed to compare cell groups transfected with either miR-143, miR-145 or miR-205 with cells transfected with the negative control duplex.

Apoptosis was measured by assessing the numbers of pre-apoptotic cells. The annexinV antibody (BD Biosciences, North Ryde, NSW) was used with propidium iodide as per the manufacturer’s protocol to stain viable, pre-apoptotic, and non-viable cells 24 and 48 h post-transfection. Propidium iodide indicated cell viability and positive AnnexinV, pre-apoptotic staining was measured using flow cytometry (BD FACSCANTO II, BD Biosciences, North Ryde, NSW) and the BD FACSDiva software package. In addition RNA was extracted from the transfected cells using *TRI*zol®, and miR-143, miR-145 and miR-205 levels were assessed in transfected cell lines using the methods described above.

### Statistical analysis

miRNA and mRNA expression data were analysed using Q-Gene software [18]. Gene expression data were tested for normality using the Shapiro-Wilk test. Differences in miRNA expression between tissues were assessed for statistical significance using the Mann–Whitney test (GraphPad Prism Software, Inc. La Jolla, CA, USA). Spearman rank order correlation tests between miRNA vs. mRNA expression in tissues were determined on-line ( http://www.wessa.net/rankcorr.wasp). Differences in proliferation and apoptosis in the Het-1A cell line were assessed using the Mann–Whitney test.

## Results

### miRNA expression and mRNA expression in tissues

Table 1 summarises the expression of the evaluated miRNA markers. The expression of miR-143, miR-145, and miR-205 were significantly higher in oesophageal squamous mucosa from subjects with ulcerative oesophagitis compared to squamous mucosa from subjects without pathological reflux. There were no significant differences between the 2 groups of squamous mucosae for the expression of miR-21, miR-194, miR-203 and miR-215.

**Table 1:** MiRNA expression in oesophageal squamous mucosa from subjects with ulcerative oesophagitis vs controls without gastro-oesophageal reflux disease

	**Controls (n=13)**	**Reflux (n=10)**	**P-value**
miR-143	0.000157	0.000361	0.0101
	(0.000171, 0.000317)	(0.000160, 0.00130)	
miR-145	0.00846	0.0177	0.0115
	(0.00458, 0.0101)	(0.00956, 0.0363)	
miR-205	0.6721	1.73	0.0026
	(0.481, 1.061)	(1.31, 2.54)	
miR-21	0.604	0.459	0.7133
	(0.304, 0.977)	(0.233, 1.25)	
miR-194	0.000138	0.000134	0.9705
	(0.000101, 0.000213)	(9.77^-05^, 0.000238)	
miR-203	0.169	0.27	0.1138
	(0.135, 0.265)	(0.206, 0.336)	
miR-215	5.99^-05^	5.45^-05^	0.7132
	(−0.000180, 0.000716)	(3.63^-5^, 0.000128)	

Relative expression values are expressed as median (95% confidence intervals).

In oesophageal squamous mucosa biopsies from individuals with ulcerative oesophagitis, there were significant positive correlations between the expression of miR-143 and *CK8* (Rho = 0.802, p = 0.004)*,* miR-143 and *BMP-4* (Rho = 0.591, p = 0.032)*,* and a significant negative correlation between miR-205 and *CK14* (Rho = −0.587, p = 0.034).

### Spatial expression of miR-143, miR-145 and miR-205 in oesophageal biopsies

**Figure 1:**
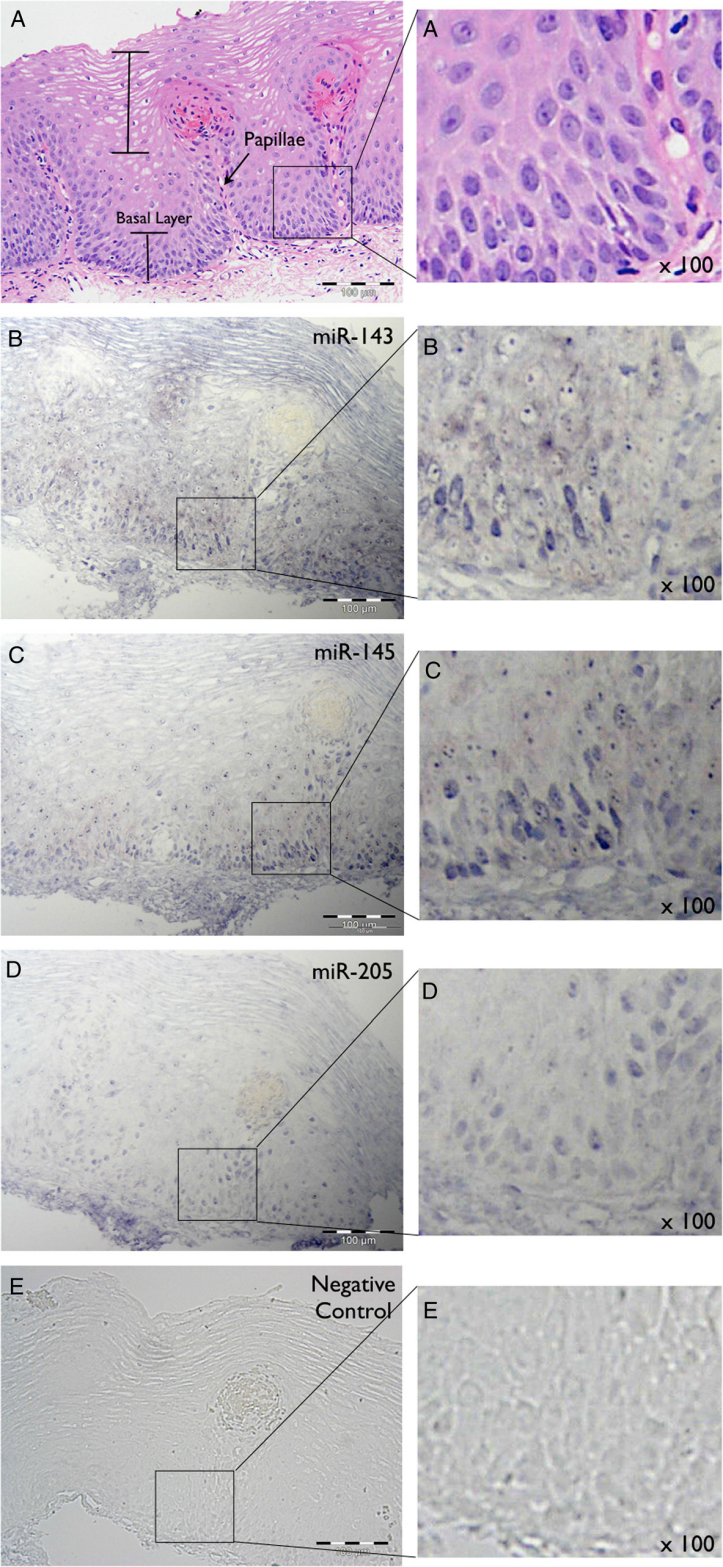
**miRNA in situ hybridisation analysis in oesophageal mucosal biopsies from patients with ulcerative oesophagitis. A:** Staining with Haematoxylin and Eosin. The basal layer, papillae and differentiated squamous epithelium are clearly visible. B, C, D and E: Hybridisation with LNA-probes for miRNAs miR-143 (**B**), miR-145 (**C**) and miR-205 (**D**), and LNA-negative control (**E**). No hybridisation was observed with the LNA-negative control probe. A region from each section has been magnified 100x. In **A**, rounded nuclei in the oesophageal epithelium are evident. **B**, **C** &**D** depict the punctate nuclear staining observed for each miRNA.

In situ hybridisation was used to study the spatial expression of miRNAs in oesophageal tissues. In oesophageal mucosal biopsies from individuals with ulcerative oesophagitis, the expression of miR-143, miR-145 and miR-205 was predominantly most intense in the basal layer of the epithelium (Figure 1). Haematoxylin and eosin stained sections showed elongated papillae and an enlarged basal layer, consistent with known gastro-oesophageal reflux histopathology (Figure 1A). Staining for miR-143 (Figure 1B), miR-145 (Figure 1C) and miR-205 probes (Figure 1D) revealed similar expression patterns, with the most intense staining seen in the basal layer of the epithelium. Staining of both the cytoplasm and nucleus was seen, with greater staining intensity seen within the nucleus in most cells. No staining was observed with the negative control duplex (Figure 1E).

### Increasing miR-143, miR-145 and miR-205 activity in Het-1A cells

**Figure 2:**
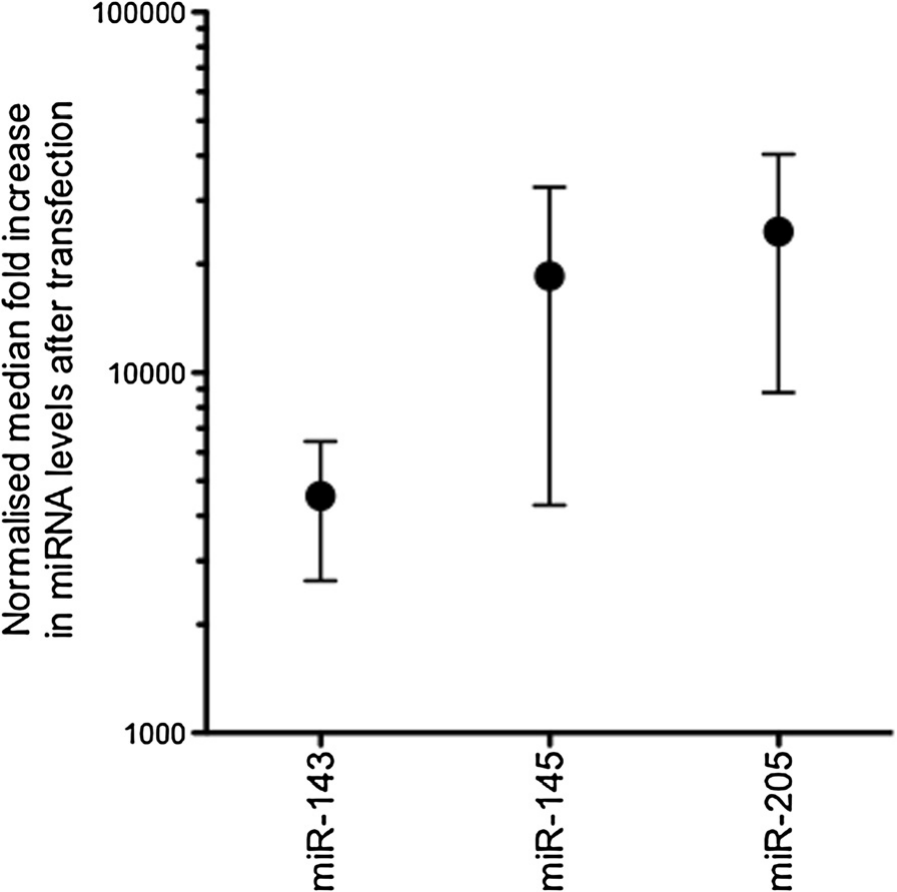
**Normalised median fold increase in miR-143, miR-145 and miR-205 levels at 24 hours after transfection with respective mimics.** The fold increase in miRNA levels was calculated as the ratio of miRNA expression in Het-1A cells transfected with a miRNA mimic compared with miRNA expression in Het-1A cells transfected with the negative control duplex.

**Figure 3:**
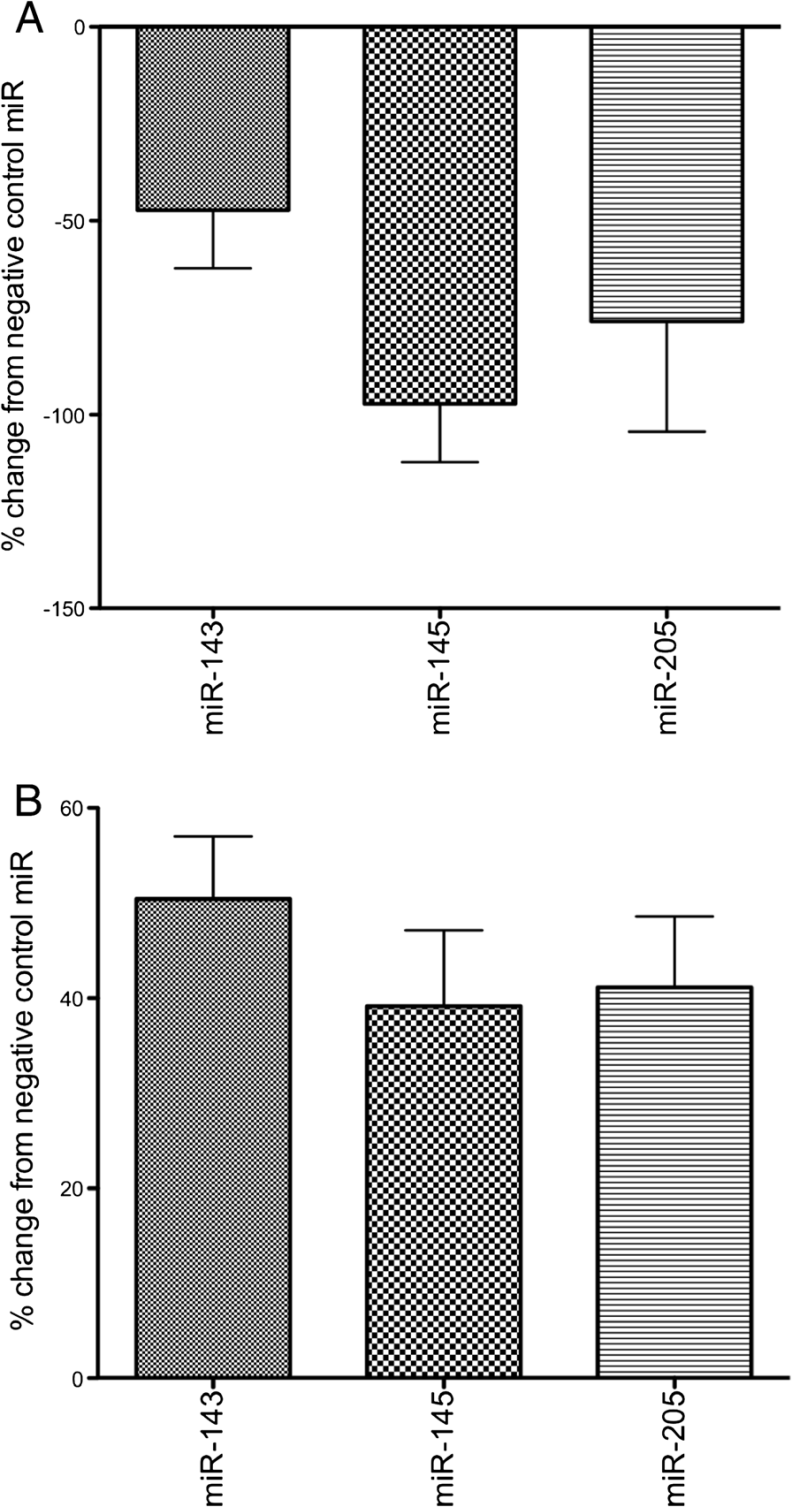
**Cell proliferation (A) and apoptosis (B) levels in Het-1A cells 48 h after transfection with miR-143, miR-145 or miR-205.** Data are represented as a percentage of the negative control. Decreased proliferation (**A**) and increased apoptosis (**B**) were observed for all miRNAs. For all negative control vs miRNA mimic comparisons p<0.05 (Mann–Whitney test).

To address the roles of the three differentially expressed miRNAs, synthetic miRNA mimics were transfected into the oesophageal keratinocyte cell line, Het-1A, to determine their effect on proliferation and apoptosis. The median fold increase in miR-143, miR-145 and miR-205 levels, 24 hours after transfection, is presented in Figure 2. Figure 3 summarises the effect of elevating miR-143, miR-145 or miR-205 activity in the Het-1A cell line. Elevated miRNA levels were associated with significantly decreased proliferation and significantly increased apoptosis.

## Discussion

miRNA regulation of gene expression has been implicated in most cellular processes [11], and it probably plays a role in the development of Barrett’s oesophagus. miRNA expression patterns might be useful biomarkers for the clinical assessment and management of Barrett’s oesophagus. Previous work from our laboratory which compared normal squamous oesophageal mucosa with Barrett’s oesophagus showed increased expression of miR-21, miR-143, miR-145, miR-194 and miR-215, and decreased expression of miR-203 and miR-205 in Barrett’s oesophagus. Ulcerative oesophagitis of the squamous mucosa, secondary to gastro-oesophageal reflux, has been shown to pre-dispose to the development of Barrett’s oesophagus [7]. Therefore, in the current study miRNAs associated with metaplasia were investigated in the earlier context of ulcerative oesophagitis.

Our study demonstrated elevated miR-143 and miR-145 levels in the oesophageal squamous mucosa from subjects with ulcerative oesophagitis (and without Barrett’s oesophagus) compared to subjects without gastro-oesophageal reflux disease. This extends our previous observation of elevated miR-143 and miR-145 expression in Barrett’s oesophagus epithelium [15], and suggests that increased expression of these miRNAs occurs prior to the development of Barrett’s oesophagus. In support of the proposal that alterations in miR-143 and miR-145 expression occur at an early stage in the development of Barrett’s oesophagus, we also observed positive correlations in expression between miR-143 and *CK8*, and miR-143 and *BMP-4*, in the oesophageal mucosa from individuals with ulcerative oesophagitis. The BMP-4 pathway is activated in the oesophageal mucosa in both ulcerative oesophagitis and Barrett’s oesophagus, and this promotes expression of columnar gene markers [9]. Further, *CK8* is a known marker of columnar epithelia [19]. Taken together, these correlations with *BMP-4* and *CK8* support an association between elevated miR-143 expression and elevated expression of columnar cell markers in reflux-exposed squamous mucosa. Whether or not miR-143 has a role in modulating gene expression programs to promote metaplastic conversion of a squamous to a columnar mucosa remains undetermined.

Although we observed upregulation of miR-143 and miR-145 in squamous mucosa from individuals with ulcerative oesophagitis, we did not observe altered expression of other miRNAs, miR-21, miR-194 and miR-215, which have previously been shown to be increased in Barrett’s oesophagus mucosa. However, support for the argument that miR-143 and miR-145 might have a role in promoting the development of Barrett’s oesophagus does not actually require the expression of other columnar miRNAs to be altered in our current study. It is important to note that the current study evaluated oesophageal squamous mucosa from individuals with ulcerative oesophagitis, and none of these individuals had Barrett’s oesophagus. Our negative findings for miR-21/194/215 are consistent with the upregulation of these miRNAs occuring later in the progression to Barrett’s oesophagus.

Two major hallmarks of oesophageal squamous mucosa in the presence of ulcerative oesophagitis include increased proliferation and apoptosis [1,3,4]. We sought to identify the spatial expression of miR-143, miR-145 and miR-205. Also, given the known association of these miRNAs with proliferation and apoptosis, we assessed possible roles in a squamous oesophageal cell line [17]. We observed reduced proliferation and increased apoptosis in the cells following transfection with miR-143, miR-145 and miR-205 mimics. Restoring miR-143, miR-145 or miR-205 expression in other cell models has also been shown to reduce cell proliferation and increase apoptosis [20-24]. In ulcerative oesophagitis miR-143, miR-145 and miR-205 staining intensity was greatest in the basal layer of the oesophageal epithelium, and it is possible that these miRNAs might direct anti-proliferative and pro-apoptotic effects within this layer. The pro-apoptotic effects of transfection with miR-143, miR-145 or miR-205 mimics may reflect the physiological apoptotic response observed in the oesophagus following reflux exposure. It is possible that up-regulation of miR-143, miR-145 or miR-205, and the associated anti-proliferative effect, might counterbalance hyperplasia in the basal layer of the oesophageal epithelium. In support of this, we identified an inverse correlation between miR-205 and *CK14*, a marker of basal cell hyperplasia and squamous restoration. Taken together, these results suggest that miR-143, miR-145 and miR-205 might suppress proliferation or promote apoptosis in the basal layer of the oesophageal epithelium. These miRNAs could be informative in studies that compare the efficacy of different treatment methods used for controlling gastro-oesophageal reflux.

Very recently, van Baal and colleagues reported that forced expression of miR-145 in Het-1A squamous oesophageal cells results in reduced cell counts and reduced expression of proliferating cell nuclear antigen [25]. However, apoptosis was not assessed in this study, and human tissues from reflux oesophagitis patients without Barrett’s oesophagus were not evaluated. Our data are consistent with the reported anti-proliferative effect of miR-145, and in addition, demonstrate apoptotic induction as a miR-145 mediated process in oesophageal squamous cells. Interestingly, this group also showed that miR-145 can modulate BMP-4 expression and alter BMP-4 signalling in Het-1A cells, thus providing *in-vitro* data suggestive of a role for miR-145 in regulating squamous to columnar cell differentiation. Taken together with our *in-vivo* observations of elevated levels of miR-145 in squamous oesophageal mucosa from individuals with ulcerative oesophagitis, the data suggest that miRNAs have a real functional role in controlling cellular identity in oesophageal mucosa in the setting of gastro-oesophageal reflux. It is possible that gastro-oesophageal reflux alters the expression of several miRNAs, including those identified in our study, in oesophageal squamous mucosa. These miRNAs could target genes and pathways that contribute to columnar metaplasia, and if enough alterations are induced this might promote the formation of Barrett’s oesophagus mucosa. Future studies could explore the miRNAs and miRNA targets involved, and use model systems to determine whether induction of a switch from squamous to Barrett’s mucosa is possible. Any model should combine both stromal and epithelial components of the oesophageal mucosa in order to faithfully replicate the complexity of cell types and signalling that is thought to be involved in Barrett’s oesophagus development [9].

In-situ hybridisation staining for miR-143, miR-145 and miR-205 appeared to be both nuclear and cytoplasmic. Nuclear staining has been reported for miR-145 [26] in breast myoepithelium, but our study is the first to show nuclear staining for miR-143, miR-145 and miR-205 in the oesophagus. Nuclear localisation of mature miRNAs is surprising, as they are typically known to exert their effects in the cytoplasm. However, recent studies also describe the nuclear localisation of mature miRNAs [27,28] and suggest that nuclear miRNAs can direct biological processes [29]. Therefore miR-143, miR-145 and miR-205 may regulate gene expression in the nucleus of oesophageal epithelial cells to exert the effects observed in our study.

Our study has some limitations. Firstly, our sample cohort was of modest size and we were not able to compare differences in miRNA expression between genders. Further validation in a larger cohort may help to identify differences in miRNA expression between genders, and may help explain why males are more likely to develop Barrett’s oesophagus than females [30]. Secondly, we limited our study to the miRNAs shown by Wijnhoven et al. [15] to be differentially expressed between normal squamous epithelia and Barrett’s oesophagus. As global miRNA expression changes were not assessed it is possible that the expression of other miRNAs may be altered in response to chronic reflux. Thirdly, we used a common transfection protocol to increase miRNA levels in Het-1A cells greater than physiological miRNA levels. This may impact on the biological relevance. However, all assay results were compared with Het-1A cells transfected with a negative control duplex at similar levels.

## Conclusions

We have shown that miRNA expression is altered in the oesophageal squamous mucosa from individuals with gastro-oesophageal reflux and ulcerative oesophagitis. These changes in miR-143, miR-145 and miR-205 expression appear to be most pronounced in the basal layer of the oesophageal epithelium. In the context of gastro-oesophageal reflux these expression changes might influence proliferation and apoptosis and thereby regulate epithelial restoration. It is reasonable to hypothesise that they could represent early molecular events preceding the development of Barrett’s oesophagus, although proving this will require further studies as described above. Future detailed analyses of the role of these miRNAs in progression from gastro-oesophageal reflux to Barrett’s oesophagus, and then to oesophageal adenocarcinoma will be valuable, and may help in efforts to control and treat these diseases.

## Competing interests

The authors declare that they have no competing interests.

## Authors’ contributions

CMS: performed the qRT-PCR miRNA experiments, performed the in-situ hybridisation experiment, and performed the cell transfection, proliferation and apoptosis assays; MZM: assisted with in-situ hybridisation and miRNA expression protocols and analyses; DIW: collection of samples; GT: assisted with sample processing and performed qRT-PCR analysis of mRNA expression; DStJA: histopathological confirmation and assessment of in-situ miRNA localisation in tissues; RH: assisted with proliferation and apoptosis experimental protocols and conducting miRNA qRT-PCR analysis; DJH: supervision of the study. CMS, MZM, DIW and DJH had lead roles in manuscript preparation, and all authors read and approved the final manuscript.

This study was funded by a Competing Project Grant from the National Health and Medical Research Council of Australia. Cameron Smith was supported by a PROBE-NET PhD scholarship funded by a Strategic research Partnerships Grant from the Cancer Council of New South Wales.

## Pre-publication history

The pre-publication history for this paper can be accessed here:

http://www.biomedcentral.com/1471-230X/13/4/prepub
